# Vessels Disturb Bottlenose Dolphin Behavior and Movement in an Active Ship Channel

**DOI:** 10.3390/ani13223441

**Published:** 2023-11-08

**Authors:** Eliza M. M. Mills, Sarah Piwetz, Dara N. Orbach

**Affiliations:** Department of Life Sciences, Texas A&M University—Corpus Christi, Corpus Christi, TX 78412, USA; eliza.m.mills@gmail.com (E.M.M.M.); sarahpiwetz@gmail.com (S.P.)

**Keywords:** anthropogenic activities, automatic identification system, behavioral state, bottlenose dolphin, Corpus Christi Ship Channel, movement, Texas, theodolite tracking, *Tursiops truncatus*, vessel activity

## Abstract

**Simple Summary:**

Dolphins alter their behavior and movement in response to human coastal activities (e.g., commercial shipping, dredging, ecotourism). The Port of Corpus Christi, Texas, is the largest port in the USA based on total revenue tonnage, yet little research has been conducted on the local bottlenose dolphins since the 1980s, prior to major oil exportation and infrastructure growth. The behavior and movement patterns of dolphins in the presence and absence of vessels were recorded using a shore-based digital theodolite and analyzed using multinomial logistic regression and generalized additive models. Dolphins frequently foraged, traveled, socialized, and milled in the Corpus Christi Ship Channel despite the presence of one or more vessels within 300 m of dolphins during 80% of observations. Dolphin behavior and movement patterns were significantly affected by season, time of day, group composition, and vessel characteristics. Dolphins appear to remain in the active Texas ship channel despite high vessel traffic. The observed dolphin–vessel interactions emphasize the need for long-term monitoring of dolphins near human activities and enforced boating regulations near important marine mammal habitats.

**Abstract:**

Although the Port of Corpus Christi, Texas, has become a top oil exporter, it is unknown if local dolphins are disturbed by high year-round vessel traffic. A shore-based digital theodolite and automatic identification system receiver were used to record data to assess common bottlenose dolphin (*Tursiops truncatus*) behavioral states and movement patterns in the Corpus Christi Ship Channel (CCSC) in relation to vessel traffic. Multinomial logistic regression and generalized additive models were applied to analyze the data. Vessels were present within 300 m of dolphins during 80% of dolphin observations. Dolphins frequently foraged (40%), traveled (24%), socialized (15%), and milled (14%), but rarely oriented against the current (7%) or rested (1% of observations). Season, time of day, group size, vessel type, vessel size, and number of vessels were significant predictors of dolphin behavioral state. Significant predictors of dolphin movement patterns included season, time of day, group size, calf presence, vessel type, and vessel numbers. The CCSC is an important foraging area for dolphins, yet the high level of industrial activity puts the dolphins at risk of human-related disturbance and injury. There is a crucial need to monitor the impact of increased anthropogenic influences on federally protected dolphins in the active CCSC, with broad application to dolphins in other ports.

## 1. Introduction

A multitude of anthropogenic activities in coastal environments (e.g., dredging, commercial fishing, recreational boating, tourism, commercial shipping) can alter marine mammal behavior and movement patterns [1,2,3]. Vessel activity poses various risks to marine mammals including short-term behavioral disruptions that reduce foraging or resting, long-term shifts in behavior that change social structure and habitat use, and collisions resulting in physical injury or death [4,5,6]. Dredging operations, tourism and recreational vessels, and the presence of many large vessels in major shipping ports and marinas contribute to increased vessel traffic and noise pollution that can change dolphin distribution and behavioral patterns [7,8,9]. Knowledge of how vessel activity impacts marine mammals is crucial as human populations continue to increase along the coast and modify marine environments [10,11,12]. Common bottlenose dolphins (*Tursiops truncatus*; henceforth “bottlenose dolphins”) demonstrating high site fidelity to a coastal area may alter their behavior or movement patterns when vessels are present (e.g., approach or avoid a vessel) [3,4,6,12,13,14,15].

Bottlenose dolphin behavioral states can be influenced by the presence of specific types of vessels, sizes of vessels, operational speeds, and distances. When multiple types and sizes of vessels were operating within 500 m of bottlenose dolphins in the Galveston Ship Channel, Texas, the dolphins foraged and socialized infrequently [3]. Bottlenose dolphin behavior in the Galveston–Bolivar ferry lane shifted from foraging to predominantly traveling when medium-sized ferries were nearby [16]. Tourism and small recreational vessels that did not adhere to regulations altered dolphin behavior and habitat use, indicating a need for proper vessel operation around bottlenose dolphins [6,16]. Dolphin-watching tours may repeatedly seek out dolphin groups in coastal areas for lengthy close-up encounters. In Koombana Bay, Western Australia, bottlenose dolphins decreased the duration of foraging, resting, and socializing and increased the duration of traveling, milling, and diving when tourism vessels were present [17]. The abundance of bottlenose dolphins declined and groups were small and spread out when tourism boats were present [6,17,18]. Compared to large slow-moving vessels, fast-moving vessels increased behavioral interruptions and avoidance responses from bottlenose dolphins [6,15,19,20]. When engine-powered vessels were present near Lampedusa Island, Italy, bottlenose dolphins reduced their feeding, resting, and socializing frequencies [15]. Milling and resting behavioral states are often sensitive to interruptions from vessels [21]. The presence of multiple vessel types and sizes in Doubtful Sound and Milford Sound, New Zealand, reduced the frequencies of socializing and resting behavioral states and resulted in habitat avoidance [14]. In South Carolina, bottlenose dolphin group sizes often decreased and behavioral states changed as vessel traffic and proximity increased [19].

Proximate encounters with vessels can also affect bottlenose dolphin movement patterns. Vessels moving at rapid speeds with unpredictable movement patterns (e.g., some small recreational vessels) may elicit evasive movements from dolphins to avoid potential vessel strikes [6]. The presence and fast approaches of recreational vessels may provoke avoidance responses from bottlenose dolphins, including increased swim speed and bearing changes as well as movement away from approaching vessels [3,4,20,22]. In the presence of engine-powered vessels, bottlenose dolphins have increased their dive duration and inter-breath intervals, thereby decreasing surface time when the potential for vessel strikes is the highest [4,22]. Slow-moving vessels with predictable movement patterns (e.g., medium and large ferries, barges, cargo carriers, and tankers) may have little or no observable disruptive effects on dolphin movement [19]. In coastal areas with high anthropogenic disturbances, vessel interactions with bottlenose dolphins need to be monitored for improved management practices of this protected species [23,24].

The Port of Corpus Christi is the largest port based on total revenue tonnage and the second-largest crude oil exporter in the USA [25]. Since a ban prohibiting oil exportation from the USA ended in 2015, the Port of Corpus Christi has had a major influx of large crude oil tankers and cargo carriers [26]. The Corpus Christi Ship Channel (CCSC) is situated on the South Texas Coastal Bend and connects the western Gulf of Mexico to the inshore Port of Corpus Christi and Corpus Christi Bay. The CCSC is being dredged to lengthen and deepen the channel to support larger commercial vessels and crude oil carriers [27,28]; however, no active dredging was observed in the study area during data collection. The increasing infrastructure in the CCSC could have detrimental effects on local wildlife and habitats.

Various vessel types are present in the CCSC throughout the year including recreational (e.g., jet ski, personal), charter, ecotourism, tug, local towing, law enforcement, trawler, ferry, barge, tanker, carrier, and cargo vessels [27]. The Port Aransas ferry operates approximately 2–6 ferries daily all year round, continually transporting vehicles across the CCSC [27]. Ecotourism vessels routinely navigate across the CCSC for wildlife viewing. Recreational vessels commonly accelerate rapidly through the CCSC between marinas and protected bays, and vessel densities in the channel may change seasonally with increased traffic in summer months, holidays, and school breaks [27]. The seasonal and daily activities of watercrafts utilizing the CCSC can affect bottlenose dolphin behavior and movement [29,30]. Previous research on common bottlenose dolphins in the CCSC, the only marine mammal species usually found in the area, last occurred in the 1980s, prior to major port growth [29,30,31]. Research is needed to understand present-day vessel effects on bottlenose dolphin behavior and movement in the CCSC. An assessment of the current interactions between bottlenose dolphins and vessels will provide insights into local dolphin sustainability as the port continues to grow and address the urgency of protecting marine mammals in areas with high vessel activity.

The objectives of this study were to: (1) track bottlenose dolphins in the CCSC–Aransas Pass confluence area; and (2) analyze how dolphin behavioral states and movement patterns vary with temporal patterns, group size, vessel characteristics, and numbers of vessels present. The behavioral states and movement patterns of bottlenose dolphins were hypothesized to vary with vessel presence.

## 2. Materials and Methods

### 2.1. Study Area

**Figure 1:**
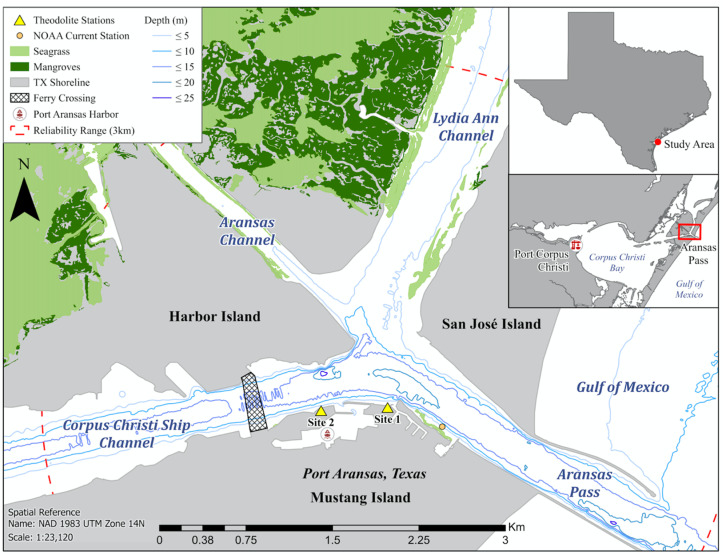
Theodolite stations (Site 1: 27°50.4867′ N, 97°3.4750′ W; Site 2: 27°50.4767′ N, 97°3.8267′ W) and marine habitats in the CCSC–Aransas Pass, Texas area.

The Corpus Christi Ship Channel (CCSC, 14.6 m deep), Lydia Ann Channel (LAC, 7.6 m deep), and Aransas Channel (AC, 4.3 m deep) are three dredged channels composed of mud and flake along the South Texas Coastal Bend [27]. The three channels converge in the study area between San José Island, Harbor Island, and Mustang Island, and connect to the Gulf of Mexico through the Aransas Pass (27°50′42.1″ N 97°03′29.1″ W; Figure 1). Channels connecting inshore and offshore environments can serve as passageways for predators and prey. The confluence area is characterized by hard engineered shorelines of concrete seawalls and granite blocks. Shorelines along the LAC and AC are lined by natural coastal habitats (e.g., sand, rocks, mangroves, seagrass) as they border undeveloped natural environments (Figure 1). The three channels support extensive daily vessel traffic, with high vessel densities and commercial vessel movements in the confluence area [27]. The entrance to the Port Aransas boat harbor extends into the confluence area with the Port Aransas ferry lane adjacent (Figure 1).

Port Aransas experiences a diurnal tide pattern with a 0.42 m range [32] and currents that average approximately 2 kn, depending on wind speed and direction [27]. Strong currents from all three channels converge in the CCSC–Aransas Pass confluence area and can make small vessel maneuverability difficult.

### 2.2. Sampling Method

Bottlenose dolphin behavioral states and movement patterns were recorded using non-invasive techniques from two elevated land-based theodolite stations during all seasons at 6 h intervals between June 2021 and September 2022. No data on bottlenose dolphins in this study were collected from a research vessel. A digital theodolite (Sokkia Model DT5/DT5S) with 30× magnification used two reference positions and a known height above sea level to triangulate the exact location of dolphins and vessels [33] (Figure 1). The theodolite eyepiece height was positioned at 24.3–24.7 m and 7–8 m above sea level for Site 1 and Site 2, respectively (Figure 1). Sampling was prioritized at Site 1 due to its elevated height and closer proximity to the CCSC–Aransas Pass confluence area. To collect geospatial positions of dolphins, the theodolite monocular crosshair was aligned at the waterline on the dolphin when it surfaced. The theodolite was connected to a Dell laptop (Inspiron 3179) with Mysticetus software (version 2021.22) to convert *x* and *y* angle measurements into Global Positioning System (GPS) coordinates in real-time and store observed dolphin behavioral states, group sizes, and vessel information. Data collection consisted of one theodolite operator and one computer operator, with the theodolite operator remaining the same individual to eliminate inter-observer bias. When searching for bottlenose dolphins, observers scanned the study area vertically from shoreline to shoreline across the CCSC.

Bushnell (12 × 50 mm) and Lakwar (10 × 50 mm) binoculars were used to initially observe dolphin behavioral states (forage, travel, social, mill, orient against current, rest; Table 1). Behavioral states were categorized as “other” and excluded from analyses when they were indistinguishable or when several occurred simultaneously. A dolphin group was defined as two or more dolphins engaged in similar behavioral states within approximately 100 m of each other [34]. Individuals or groups of dolphins were actively tracked with the theodolite for approximately 40 min every hour, and their positions, group sizes, and behavioral states were recorded. Dolphin groups included adults and calves (echelon swimming position and <2/3 the size of an adult) [35]. At the beginning of each survey day, the largest group of dolphins observed were tracked first, then the second largest, and so on. Data were collected approximately every other time the tracked dolphin or majority of the group surfaced to obtain a precise location measurement. When individuals in a group were spread out, the approximate middle of the group was marked, while the position of the middle individual was marked when dolphins were close together in a group. Dolphins were tracked until an individual or group was lost, visibility was restricted from environmental conditions (e.g., sunset, fog, Beaufort state > 3), tracking time exceeded 1 h, or individuals/groups moved beyond the reliable visibility range of the theodolite (>3 km; Figure 1) [36].

Dolphin swimming speed (m/s) and vessel speed (m/s) were automatically calculated in Mysticetus software for each dolphin and vessel position recorded. Swimming speed was calculated by taking the total distance traveled between two consecutive dolphin positions and dividing by the time lapsed for the dolphin to move between the selected positions. Vessel speed was calculated the same way. Dolphins with swimming speeds ≤11 m/s and vessels with travel speeds <25 m/s were included in the analyses, while those with greater speeds were unrealistic and filtered out. The bearing of a dolphin position is the angle between a meridian on Earth and the line connecting two consecutive dolphin points [41]. The bearing (degrees) was calculated based on the great-circle arc using the two consecutive geographic coordinates from the same group of dolphins. Change in bearing, the absolute value in degrees bound by 0 and 180, was calculated *post hoc* by subtracting one bearing from the preceding bearing based on 3 consecutive geographic coordinates from the same group of dolphins. Larger or greater bearing changes constituted large changes in swim direction or less linear swim paths, while smaller or minimal bearing changes indicated small changes in swim direction or more linear swim paths.

Geographic positions or tracks and speeds of commercial vessels (i.e., barges, cargo carriers, charters, ferries, law enforcement, tankers, tugs) with automatic identification system (AIS) signatures were automatically recorded onto Mysticetus at 30 s intervals through a dual channel Matsutec AR-10 USB receiver. Vessel type and approximate size of vessels with AIS signatures were obtained *post hoc* from recorded AIS tracks using Maritime Mobile Service Identity numbers and Marine Traffic [42]. Vessels observed near dolphins without AIS (i.e., recreational vessels, ecotourism vessels) were tracked by marking at least two theodolite positions at the waterline along the hull, which enabled Mysticetus to automatically calculate vessel speed (m/s). Vessel type and categorical size (small, medium, large, extra-large; Table 2) were recorded in Mysticetus for each vessel tracked by theodolite. All vessels were categorized *post hoc* by type, size, and mean speed (Table 2). Barges and tank-barges were categorized as coastal cargo ships, charters and trawlers were categorized as fishing vessels, extra-large carriers and tankers were categorized as offshore commercial ships, law enforcement and port tenders were categorized as enforcement, and inshore and offshore supply vessels were categorized as supply vessels.

All vessel positions recorded within 300 m and 5 min of each dolphin position were selected and paired with the corresponding dolphin position for analyses [6]. When multiple vessels were paired with a single dolphin position, the number of vessels was totaled, the vessel type and size were defined as “mixed” if multiple categories were present, and the speed (m/s) and distance (m) of the vessel nearest to the paired dolphin were used in analyses. Vessels were categorized as absent when no vessel was recorded within 300 m and 5 min of a dolphin position; thus, vessel type and size were defined as “none”.

Seasons were defined as summer (June–August), fall (September–November), winter (December–February), and spring (March–May). Sampling occurred throughout the day, categorized into morning (800–1059 h), midday (1100–1359 h), early afternoon (1400–1659 h), and late afternoon (1700–2000 h).

### 2.3. Analyses

Statistical software RStudio (version 2022.07.2) was used to analyze dolphin behavioral and movement patterns relative to vessels. A multinomial logistic regression (MLR) using the “nnet” package was used to analyze the relationship between behavioral states and predictor variables [43,44,45]. The eight predictor variables were categorical (season, time of day, vessel type, vessel size) and numerical (group size, vessel speed, number of vessels, and vessel distance from dolphin(s)). Travel was used as the behavioral reference as it involves consistent movement and occurred often. Summer and midday were the temporal references as most collection effort occurred during those times. Vessel type “none” and vessel size “none” were used as reference groups as they indicated vessel absence. An augmented pairs plot was used to assess potential collinearity between predictor variables and variables demonstrating multicollinearity, and the less explicable variable was removed from the model. MLR models were compared by stepwise removal of predictor variables based on Akaike Information Criterion (AIC) from the fully saturated model to a proposed model [46]. The best-fitting model was selected with the lowest AIC and deviance [46,47]. The final MLR model’s goodness of fit of the observed data with the expected results was tested using a Pearson’s chi-squared test and explained with Nagelkerke Pseudo-R-square test. Log-likelihood ratio tests were used to determine the significance of categorical predictor variables on behavioral state in the model. Contingency tables showed the relationship between significant predictor variables and behavioral state [47]. The “multcomp” package in RStudio was used to perform a one-way analysis of variance (ANOVA) and a *post hoc* Tukey test with Shaffer adjustment on significant numerical predictor variables.

Generalized additive models (GAMs) were used in RStudio with the “mgcv” package to analyze potential explanatory variables for swim speed and bearing change [48,49,50]. There were nine potential explanatory categorical variables (season, time of day, vessel type, vessel size) and numerical variables (group size, number of calves, vessel speed, number of vessels present, and distance of nearest vessel to dolphin(s)). Shapiro–Wilk normality tests (*a* = 0.05) were used to determine if the data were distributed normally. Collinearity among potential explanatory variables was assessed with augmented pairs plots and variance inflation factor (VIF) values. If collinearity was detected between potential explanatory variables, the less explicable variable was removed from the model. Smoothing terms enabled model flexibility of the GAM by assigning the number of knots for smooth terms in the model [48,51]. The default of 10 knots was used unless smoothed variables contained less than 10 levels, in which case knot selection was reduced. The condition for removing smooth terms, smoothing functions, and linear terms from the full model included a decrease in the generalized cross-validation (GCV) score, an increase in the *R*^2^-adjusted value, and an increase in the deviance-explained percentage [48]. If these conditions were not met, the terms were retained in the model. Linear mixed-effects modelling, using the lme function (package “nlme”), was run to detect temporal autocorrelation of residuals in the selected models. If a highly significant correlation was detected, autoregressive models AR(1) and ARMA were applied to improve the autocorrelation plots, or data were filtered based on the most significant lag positions.

## 3. Results

Data were collected from theodolite stations in Port Aransas for 63 days and 287.8 h from June 2021 to September 2022. Data collection occurred during all seasons (summer: *n* = 124.6 h, mean 41.5 h/month; fall: *n* = 61.7 h, mean 20.6 h/month; winter: *n* = 40.7 h, mean 13.6 h/month; spring: *n* = 60.8 h, mean 20.2 h/month) and times of day (morning: *n* = 51.5 h, mean 1.4 h/day; midday: *n* = 112.3 h, mean 2.0 h/day; early afternoon: *n* = 81.9 h, mean 1.7 h/day; late afternoon: *n* = 24.9 h, mean 1.8 h/day). Most data collection effort occurred during the summer months and during the midday period (Table S1). A total of 6189 positions of dolphins were collected and used in various analyses: 4339 positions were used in behavioral analyses, 4018 positions were used in swimming speed analyses, and 4372 positions were used in bearing-change analyses. Foraging (*n* = 1869 positions, 40% of tracks) was the primary dolphin behavioral state in the CCSC–Aransas Pass confluence area, followed by traveling (*n* = 1127 positions, 24% of tracks), socializing (*n* = 683 positions, 15% of tracks), milling (*n* = 660 positions, 14% of tracks), orienting against current (*n* = 316 positions, 7% of tracks), and resting (*n* = 45 positions, 1% of tracks).

A total of 644 dolphin groups were tracked with a daily mean (±SD) of 10.2 ± 4.2 groups (range = 3–28 groups). Group sizes ranged from 1 to 25 dolphins (mean ± SD adults = 4.58 ± 3.0) with 0–4 calves present (mean ± SD calves = 0.17 ± 0.5). Dolphins were not individually identified; thus, the total number of adults (2949) and calves (112) may be smaller due to potential resightings of a dolphin across groups.

A total of 6584 vessels were tracked with a daily mean (±SD) of 105 ± 40.6 vessels (range = 17–241 vessels). Vessel presence varied seasonally (summer: *n* = 2744, mean = 22.0 vessels/h; fall: *n* = 1571, mean = 25.5 vessels/h; winter: *n* = 920, mean = 22.6 vessels/h; spring: *n* = 1349, mean = 22.2 vessels/h) and with time of day (morning: *n* = 1595, mean = 30.9 vessels/h; midday: *n* = 2594, mean = 23.1 vessels/h; early afternoon: *n* = 1808, mean = 22.1 vessels/h; late afternoon: *n* = 587, mean = 23.6 vessels/h). Eighty percent of tracked dolphin positions had vessels present within 300 m and 5 min. On average (±SD), 2 ± 1.7 vessels (range = 0–12 vessels) were present with each dolphin group. A total of 1318 vessels were within 300 m and 5 min of each dolphin position and subsequently used in analyses.

### 3.1. Behavioral Patterns

The fully saturated MLR model overpredicted the more common behavioral states and masked the least common behavioral states (orient against current, rest); therefore, the least common behavioral states were excluded to improve the final MLR model. The final MLR model included the four most common behavioral states (forage, travel, social, mill; Table 3). Vessel distance to dolphins(s) was excluded from the analysis due to multicollinearity, and vessel speed was excluded based on AIC values. The best-fitting model for behavioral state (*n* = 4339 positions, AIC = 10,424.16, deviance = 10,286.16) included the significant predictor variables of season, time of day, group size, vessel type, vessel size, and number of vessels present (X^2^_9_ = 405.14, *p* < 0.001; Table 4). However, the Nagelkerke pseudo-R-square was 21%, showing a weak goodness of fit between behavioral states and predictor variables. The patterns described for frequencies of occurrence refer to statistical trends of occurrences expected by chance.

Dolphins foraged frequently in the winter in the morning and late afternoon and infrequently in the summer and early afternoon (Table S2 and Table S3). Traveling frequently occurred in the early afternoon and infrequently in the spring and late afternoon, while milling occurred frequently in the summer (Table S2 and Table S3). Dolphins socialized extensively in the spring with little socialization in the winter (Table S2). Milling and socializing occurred in similar amounts across all times of day (Table S3).

A statistically significant difference was found in dolphin group size across behavioral states (*F*(3, 4339) = 67.49, *p* < 0.001), with large groups engaged more in foraging (*t* = 6.23, *p* < 0.001), socializing (*t* = 12.22, *p* < 0.001), and milling (*t* = 10.96, *p* < 0.001) than traveling. Dolphin group sizes were not significantly different when socializing or milling (*t* = 1.01, *p* > 0.05), yet larger when compared to foraging (*t* = 8.00, *p* < 0.001; *t* = 6.68, *p* < 0.001, respectively).

**Figure 2:**
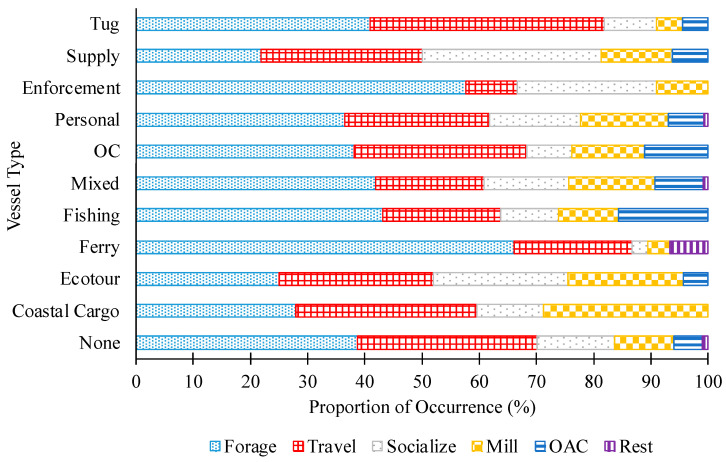
Proportion of occurrence of common bottlenose dolphin behavioral states paired with vessel types in the CCSC–Aransas Pass, Texas area (*n* = 4339 dolphin positions). Vessel types: mixed (*n* = 1858), personal (*n* = 1101), none (*n* = 939), ecotour (*n* = 208), ferry (*n* = 180), fishing (*n* = 160), coastal cargo (*n* = 104), OC = offshore commercial (*n* = 63), enforcement (*n* = 33), supply (*n* = 32), tug (*n* = 22).

Most dolphins were tracked when there were “mixed” (multiple) vessel types and “mixed” vessel sizes within 300 m and 5 min of a dolphin position (Figure 2; 39 and 38% of positions, respectively). Dolphins foraged particularly frequently near ferries, fishing vessels, and enforcement vessels (Figure 2 and Table S4) and near vessels of mixed sizes, yet infrequently near small sized vessels (Table S5). Traveling frequently occurred when supply vessels and tugs were nearby (Figure 2; Table S4) and when no vessels were present, yet infrequently when mixed vessel types and sizes were nearby (Table S5). Dolphins foraged, traveled, and socialized in similar proportions when ecotourism vessels were proximate (Figure 2). Socializing occurred more than other behavioral states when supply vessels (Figure 2; Table S4) and small vessel sizes were nearby (Table S5). Milling occurred frequently when coastal cargo ships and small vessel sizes were nearby and infrequently when ferries were nearby or no vessels were present (Figure 2; Table S4 and Table S5).

There was a statistically significant difference in the number of vessels present across behavioral states (*F*(3, 4339) = 23.49, *p* < 0.001); when many vessels were present, dolphins foraged (*t* = 7.93, *p* < 0.001), milled, (*t* = 6.23, *p* < 0.001), and socialized (*t* = 4.31, *p* < 0.001) more often compared to traveling. There were no significant differences associated with the number of vessels present and dolphins engaged in milling and foraging (*t* = 0.14, *p* > 0.05), socializing and foraging (*t* = −2.01, *p* > 0.05), and socializing and milling (*t* = −1.77, *p* > 0.05).

### 3.2. Swimming Speed and Bearing-Change Patterns

Autocorrelation existed between swimming-speed model residuals, thus the second data point for each focal individual/group was removed. The application of autoregressive models AR(1) and ARMA to the swimming-speed and bearing-change data did not improve the autocorrelation plots. Swimming speed (W = 0.717, *p* < 0.001) and bearing change (W = 0.934, *p* < 0.001) were not normally distributed; however, the sample size is robust (*n* = 4018 and 4372 positions, respectively). Generalized additive models (GAMs) used the quasi-likelihood family in which the distribution assumption can be relaxed [52]. Due to multicollinearity, vessel size, speed, and distance to dolphin(s) were excluded. The best-fitting GAM describing significant variations in swimming speed (adj-R^2^ = 0.0602, GCV = 0.454, deviance explained = 6.55%) including explanatory smooth terms (group size, number of calves) and linear terms (season, time, vessel type, number of vessels present) is:

[*gam*(*SwimSpeed*)~*s*(*GroupSize*, *k* = 4) + *s*(*Calves*, *k* = 4) + *Season* + *Time* + *VesselType* + *VesselNumber*]

The best-fitting GAM describing significant variations in bearing change (adj-R^2^ = 0.065, GCV = 3690, deviance explained = 7.01%) including explanatory smooth terms (number of vessels present, group size, number of calves) and linear terms (season, time, vessel type) is:

[*gam*(*BearingChange*)~*s*(*VesselNumber*) + *s*(*GroupSize*) + *s*(*Calves*, *k* = 4) + *Season* + *Time* + *VesselType*]

**Figure 3:**
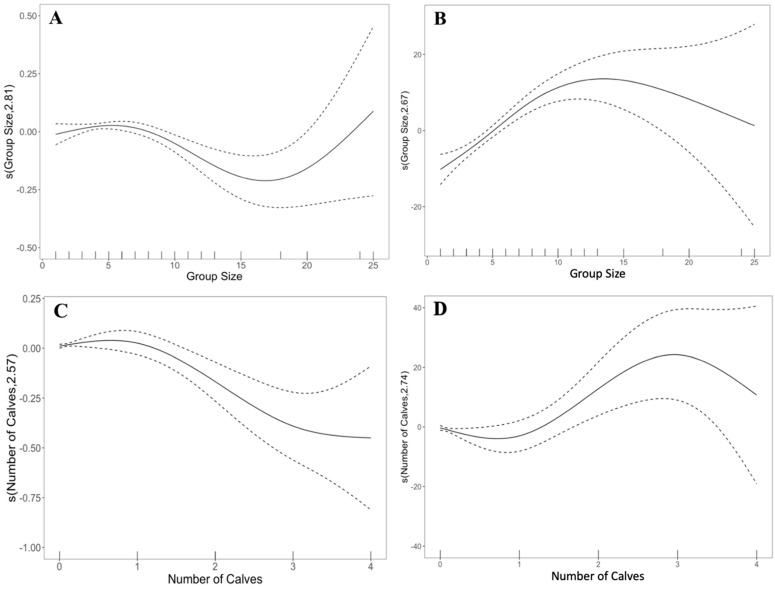

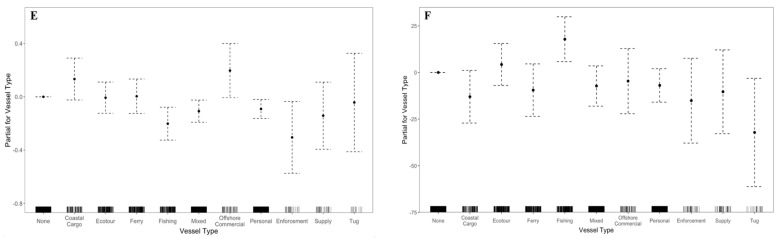
Some significant explanatory variables in the best-fitting generalized additive model for common bottlenose dolphin swimming speed (**left**) and bearing change (**right**) include (**A**,**B**) group size, (**C**,**D**) number of calves, and (**E**,**F**) vessel type. R output includes predicted smoothing functions for continuous explanatory variables, and a partial dependence plot for the categorical predictors. Scaling of the *y*-axis varies among predictor variables to emphasize model fit; the estimated degrees of freedom for smooth terms are in brackets. Correlations with swim speed and bearing change: positive (values > 0), negative (values < 0), no effect (0 value). Dashed lines for smooth and linear terms indicates 95% confidence intervals. Vertical hashmarks along the *x*-axis illustrate sample size.

Swimming speed was slower across all seasons compared to the summer (fall: *t* = −3.27, *p* < 0.01; spring: *t* = −9.73, *p* < 0.001; winter: *t* = −9.664, *p* < 0.001), while bearing change was higher (fall: *t* = 4.92, *p* < 0.001; spring: *t* = 5.97, *p* < 0.001; winter: *t* = 9.40, *p* < 0.001). Swimming speed was slower during the morning (*t* = −2.56, *p* < 0.05) and late afternoon (*t* = −3.68, *p* < 0.001) compared to midday, but not the early afternoon (*t* = −0.932, *p* > 0.05). In contrast, bearing change was higher during the late afternoon (*t* = 4.96, *p* < 0.001) compared to midday, but not the morning (*t* = 1.37, *p* > 0.05) nor early afternoon (*t* = −0.79, *p* > 0.05). Swimming speed decreased (*F* = 4.96, edf = 2.81, *p* < 0.01) and bearing change increased (*F* = 14.83, edf = 2.67, *p* < 0.001) with increasing group sizes until there were 15 dolphins, then patterns reversed when >15 dolphins were present (Figure 3A,B). Swimming speed decreased as the number of calves present increased (*F* = 7.39, edf = 2.57, *p* < 0.001; Figure 3C), while bearing change increased (*F* = 3.87, edf = 2.74, *p* < 0.05) until three calves were present and then decreased (Figure 3D). Swimming speed (*t* = 2.31, *p* < 0.05) and bearing change increased (*F* = 9.48, edf = 2.40, *p* < 0.001) with the number of vessels present. Swimming speeds were slow when fishing (*t* = −3.21, *p* < 0.01), mixed (*t* = −2.54, *p* < 0.05), personal (*t* = −2.52, *p* < 0.05), and enforcement vessels (*t* = −2.22, *p* < 0.05) were proximate to dolphins (Figure 3E). Bearing changes were low near tug vessels (*t* = −2.17, *p* < 0.05) yet high near fishing vessels (*t* = 2.91, *p* < 0.01; Figure 3F). Other vessel types had no statistically significant effect on dolphin swim speeds or bearing changes.

## 4. Discussion

Bottlenose dolphins in the CCSC–Aransas Pass confluence area consistently encounter heavy vessel traffic all year round; this study shows that >20 vessels per hour typically pass through the area with a daily mean of 105 vessels present during daylight hours. Vessels were proximate to 80% of dolphin groups, with two vessels near each group on average, and this anthropogenic disturbance appears to have substantial effects on dolphin behavioral states and movement patterns. Vessels of mixed sizes were near dolphins most often, supporting that a variety of vessel types and sizes pass through the CCSC daily [28]. Despite high vessel traffic, bottlenose dolphins consistently use the industrialized CCSC–Aransas Pass confluence area primarily to forage, travel, socialize, and mill, with little orienting against current or resting occurring.

Dolphins foraged most in the winter, consistent with historical movement of prey species through Texas passes in the winter and early spring (flounder, *Paralichthys lethostigma, P. albigutta* [53]; red drum, *Sciaenops ocellatus* [54]; spotted seatrout, *Cynoscion nebulosus* [55]). High occurrences of dolphin foraging in the morning and late afternoon and low occurrences in the early afternoon are congruent with other studies along the Texas coast [3,37], and are consistent with this study’s findings of slow swimming speeds in morning and late afternoon. Frequent changes in direction and slow swimming while foraging may reflect the coordinated herding of prey [56]. Thus, the comparatively fast swimming speeds and linear swimming paths of dolphins in the summer are consistent with reduced foraging during the summer and may reflect reduced prey availability. Although some prey species leave the CCSC and move into the Texas bays in the spring and summer [53,54,57], dolphins remained present in the CCSC during these seasons with reduced traveling and increased milling. High occurrences of traveling in the early afternoon are consistent with other studies [3,33]. The high occurrences of socializing in the spring, which is a behavioral state associated with slow swimming speeds and high bearing changes, may be indicative of mating as bottlenose dolphins have a 12-month gestation period and there were high numbers of calf sightings in the spring (April and May) [29]. As the mean number of vessels present per hour was highest in the fall and during the morning and late afternoon, dolphins may swim slowly in non-linear paths (i.e., many bearing changes) during these periods to avoid extensive vessel traffic and potential vessel strikes [4,6,22].

Large group sizes were associated with foraging, socializing, and milling behavioral states. As three channels converge in the CCSC–Aransas Pass area before flowing into the Gulf of Mexico, dolphins may feed, mate, and play in large groups before traveling out of the area in smaller groups. Dolphin group sizes were smaller when foraging compared to milling and socializing potentially because larger groups may reduce the net yield per individual [38]. Dolphins swam slower and increased directional changes with increasing group size, possibly as a protective response to vessel traffic in the CCSC, especially when calves are present. The increased presence of calves may drive slow group swim speeds and large directional changes, possibly for mothers to maintain contact with calves while avoiding vessel activity [58]. Calves have been observed in the CCSC–Aransas Pass confluence area during every month of the year and mother–calf pairs have been described in other studies as fusing with groups rather than remaining alone [29]. As most dolphin groups in this study were small (averaging 5 individuals) with no calves present (averaging <1 calf per group), the fast swim speeds and decreased directional changes identified with larger groups (>15 individuals) or with additional calves (>3 calves) may not indicate accurate movement patterns.

Vessel activity influenced all dolphin behavioral states in the CCSC. Dolphins frequently foraged in the presence of multiple vessel sizes in contrast to dolphins in the Galveston Ship Channel, Texas, that foraged infrequently when vessels were present [3,16]. The collective movement of vessels of various sizes may aid in mixing surface currents and nutrients, increasing prey abundance, or displacing prey. Foraging occurred frequently when the ferry was proximate to dolphins, similar to another study in Texas waters [16]. The mixing of nutrients and currents from consistent ferry movement and the channel confluence area may increase prey abundance in ferry lanes. Although infrequently observed (1% of observations), resting occurred most often when ferries were near dolphins. Dolphins may rest along the channel periphery between bouts of foraging near the Port Aransas ferry; however, resting behavior should be interpreted with caution given the low sample size. Low occurrences of resting behavior during the daytime have been documented in the Patos Lagoon estuary, Brazil, an area of high vessel activity [58], and increased resting may occur at night [59] in the absence of vessel traffic [3,15,17]. While OAC was infrequently observed, most occurrences were near fishing vessels where frequent bearing changes and traditional foraging occurred, supporting the hypothesis that OAC may be a foraging specialization in the dredged CCSC [39].

While bottlenose dolphins appear to vary swim speed and bearing change with vessel type, dolphins swam quickly with less linear paths when many vessels were present, possibly to avoid potential vessel strikes. Traveling involves linear movement between locations. Traveling frequently occurred when supply vessels and tugs were nearby, possibly because their slow and predictable movements may reduce the perceived injury risk to dolphins. The low frequency of bearing changes near tug vessels further supports that dolphins were traveling in a fast linear direction. Traveling also occurred frequently when no vessels were present. Traveling often occurred frequently in other industrialized areas when vessels were present [16,17]. High vessel traffic in the CCSC–Aransas Pass confluence area could interrupt linear dolphin travel between channels, prompting dolphins to travel more when vessels are absent and potentially mill frequently with high vessel activity [17]. Dolphins milled less than expected by chance when no vessels were present yet frequently when coastal cargo and small vessels were present. The increased maneuverability of small vessels allows them to move in fast, unpredictable patterns, potentially prompting evasive movements from dolphins to avoid collisions [6]. In the CCSC, dolphins may mill often with small vessel presence, as the frequent heading changes and nondirectional movement characterized as milling may be a response to unpredictable small vessel operation. Socializing occurred more than other behavioral states when supply vessels and small vessel sizes were nearby. Dolphins in close proximity may initially attract small vessels to observe them. Contrary to previous studies [15,17], dolphins in the CCSC appeared to socialize in the presence of small vessels (e.g., ecotourism, personal recreational); however, it is undetermined whether dolphins decrease social behavior during vessel approaches or engage in avoidance responses near fast-moving vessels as observed elsewhere [6,15,19,20]. Dolphin-watching ecotourism vessels were often observed closely following dolphin groups in the CCSC. Dolphins swam slowly with large changes in bearing, engaging in similar proportions of foraging, travelling, and socializing. Although ecotourism vessels can alter dolphin abundance, behavior, and movement [6,17,18], dolphins may also become habituated, desensitized, or tolerant and exhibit no external response to vessel presence [16,19,60,61]. For similar reasons, dolphins may have swum slowly in the presence of fishing, mixed, enforcement, and personal vessel types, all of which move fast and unpredictably. Previous studies showed that small vessels moving at fast speeds interrupted dolphin behavioral states [6,15,19,20] and increased dolphin swimming speeds and changes in direction [3,4,6,20,22].

The number of vessels present consistently has effects on dolphin behavioral states and movement patterns [3,4,19,20,22,62]. Up to 12 vessels were observed with a single dolphin group. Dolphins swam faster and had greater bearing changes as the number of vessels increased. Vessel activity in the CCSC–Aransas Pass area is extensive and has been categorized by the U.S. Coast Guard as one of the most dangerous areas for vessel traffic as it is a blind spot for large vessels [27]. However, bottlenose dolphins continue to use the confluence area to forage, socialize, and mill with large numbers of vessels present, indicating that dolphins may be habituated or desensitized to high volumes of vessel traffic [16,60,61]. Dolphins may remain in the CCSC–Aransas Pass confluence area due to high prey abundance despite potential risks of vessel strikes.

This study provides insights into bottlenose dolphin behavior and movement patterns during vessel interactions in the CCSC. Vessel operations in close proximity to dolphins may lead to long-term behavioral changes that impact social structure and habitat use or cause injuries and death to dolphins [4,6,17]. We recommend that future studies explore how vessel speed affects dolphins. Continued studies on dolphin behavior, group composition and dynamics, and movement patterns in the CCSC are vital to monitor anthropogenic impacts to dolphins as coastal operations in Corpus Christi and Port Aransas are ongoing. For example, the effects of vessels on dolphin groups with and without calves may yield further insights into evasion and habituation to vessels. Fine-scale responses of dolphins to vessel presence (e.g., diving behavior, acoustic communication, duration of dolphin and vessel interactions, variation in distances between vessel operations and dolphins, vessel speed approaches and directions) will improve understanding of the effects of vessel activity on dolphins in the CCSC. Long-term monitoring projects of vessel impacts on dolphins have led to management practice and policy changes including the establishment of speed restriction zones [23], curtailed numbers and duration of daily dolphin-watching ecotourism trips [62], and enforced defined vessel approach distances to dolphins [24]. As laws for boating around dolphins in Texas are currently lacking, extensive knowledge of the impacts of vessel operations on Texas dolphins could prompt the development of boating regulations near dolphins to better protect the species. Vessel activity and infrastructure development in the CCSC–Aransas Pass area have increased substantially in recent decades, with dolphins possibly habituated or desensitized to disturbances. Long-term monitoring of bottlenose dolphins in the area is recommended to understand dolphin abundance, occurrence, site fidelity, habitat use, and response to increasing anthropogenic operations. Knowledge of dolphin behavior and movement patterns near vessels can prompt sustainable planning efforts to minimize the long-term health impacts of anthropogenic disturbances on coastal Texas dolphins.

## 5. Conclusions

Bottlenose dolphins foraged, traveled, socialized, and milled across all seasons and times of day in the CCSC–Aransas Pass confluence area, despite high vessel traffic, dredging, and marine construction. The quantity of vessels varied seasonally and across times of day and are known to affect dolphin behavior and movement in the area [29,30]. Most (80%) dolphins were observed with vessels present and swam faster in less linear paths as the number of vessels increased. While vessel presence > 300 m from dolphins was not assessed, distant vessels may effect dolphin behavior and movement in the CCSC as acoustic signals travel far. Foraging often occurred near ferries and dolphins may rest between bouts of foraging. Resting was observed infrequently as dolphins may leave the area to rest away from high volumes of vessel traffic. Contrary to previous studies [6,16,17], foraging, traveling, and socializing occurred when ecotour vessels were present, suggesting that dolphins may be desensitized or tolerant of dolphin-watching tours; however, this study did not determine pre- and post-tour responses. Insight into interactions between vessels and dolphins could highlight the urgency for continued monitoring of anthropogenic activities and the creation of regulations for proper vessel operation near dolphins. As humans continue to use marine environments, it is important to understand the impacts anthropogenic activities have on coastal species. As the Port of Corpus Christi continues to play its part in oil exportation and proceeds with infrastructure growth to support additional and larger vessels, investigating how dolphin behavior and movement patterns vary with temporal patterns, group size, and vessel interactions is necessary to inform the management of support for species protection.

## Figures and Tables

**Table 1:** Behavioral states of common bottlenose dolphins observed in the CCSC–Aransas Pass, Texas area from June 2021 to September 2022. Descriptions are adapted from various published literature.

Behavioral State	Description	Source
Forage	Variable movement directions, high arching dives (tail flukes out of water), interacting with fish (trapping fish against hard structures)	[37,38]
Mill	Nondirectional movement, absence of physical contact, frequent changes in heading	[37,39,40]
Orient against current (OAC)	Frequent surfacing, no position change, oriented against a visible current	[29], termed “rest” [39], “forage”
Rest	Slow movement, drifting in one direction at the surface	[39]
Socialize	Individuals in close proximity, body contact, sexual behavior, leaps, playing with objects	[29,40]
Travel	Steady or rapid movement in one direction	[37]

**Table 2:** Vessel type, size (m), and mean speed (m/s ± SD) categories in the CCSC–Aransas Pass, Texas area (*n* = 65,026 vessel positions).

Vessel Type	Vessel Size (m)	Mean Speed (m/s)
Ecotour Ferry Fishing (charter) Personal recreational Enforcement (law enforce) Tug	Small (<10)	4.02 ± (5.02)
Coastal cargo (barge) Ecotour Ferry Fishing (charter, trawler) Personal recreational Enforcement (law enforce, port tender) Supply (inshore, offshore) Tug	Medium (10–30)	1.96 ± (1.32)
Coastal cargo (barge, tank-barge) Ferry Fishing (trawler) Supply (offshore) Tug	Large (31–70)	1.42 ± (0.98)
Coastal cargo (barge) Offshore commercial (carrier, tanker) Tug	Extra-Large (>70)	2.47 ± (0.69)

**Table 3:** Exponentiated coefficients (±SE) of the best-fitting final multinomial logistic regression model evaluating the relationship between common bottlenose dolphin behavioral states (forage, travel, social, mill) and temporal patterns, dolphin group size, and vessel characteristic predictor variables (*n* = 4339). Standard error values are included in parentheses. Multinomial logistic regression reference groups (travel, summer, midday, vessel type and size none) are not included.

Independent Variables		Behavioral States		
		Forage	Mill	Social
Season	Fall	1.27 (0.10)	0.45 (0.14)	1.68 (0.14)
	Spring	1.95 (0.11)	0.81 (0.15)	3.34 (0.14)
	Winter	4.07 (0.14)	0.75 (0.20)	1.38 (0.22)
Time of day	Morning	1.76 (0.12)	1.08 (0.15)	1.05 (0.16)
	Early PM	0.73 (0.09)	0.67 (0.12)	1.12 (0.12)
	Late PM	3.66 (0.17)	1.42 (0.22)	2.47 (0.22)
Group size	––	1.10 (0.01)	1.17 (0.02)	1.20 (0.02)
Vessel Type	Coastal cargo	0.43 (0.31)	1.58 (0.34)	1.14 (0.41)
	Ecotour	0.98 (0.25)	1.42 (0.30)	1.87 (0.31)
	Ferry	2.15 (0.26)	0.55 (0.45)	0.25 (0.51)
	Fishing	1.51 (0.24)	1.05 (0.34)	0.84 (0.35)
	Mixed	1.36 (0.21)	1.83 (0.27)	1.23 (0.29)
	Offshore commercial	0.75 (0.57)	0.93 (0.65)	0.60 (0.83)
	Personal	1.07 (0.22)	1.21 (0.28)	0.85 (0.30)
	Enforcement	2.05 (0.63)	1.35 (0.81)	4.45 (0.71)
	Supply vessels	0.34 (0.51)	0.95 (0.62)	1.27 (0.51)
	Tug	0.55 (0.53)	0.34 (1.07)	0.28 (0.86)
Vessel size	Small	0.68 (0.21)	1.22 (0.28)	1.15 (0.29)
	Medium	1.16 (0.21)	1.34 (0.27)	0.70 (0.29)
	Large	1.19 (0.35)	0.92 (0.56)	1.19 (0.53)
	Extra large	1.13 (0.49)	1.32 (0.52)	0.72 (0.69)
	Mixed	0.54 (0.22)	0.60 (0.30)	0.64 (0.31)
Number of vessels	––	1.12 (0.03)	1.17 (0.04)	1.10 (0.04)

**Table 4:** Log-likelihood ratios (*a* = 0.05) of significant predictor variables included in the best-fitting final multinomial logistic regression model (*n* = 4339).

Predictor	Log-Likelihood	X^2^	df	Pr (>X^2^)
Season	−5305.80	325.48	−9	<2.2 × 10^−16^
Time of day	−5228.80	171.46	−9	<2.2 × 10^−16^
Group size	−5224.40	162.68	−3	<2.2 × 10^−16^
Vessel type	−5198.80	111.52	−27	0.0000
Vessel size	−5155.10	24.07	−12	0.0199
Number of vessels	−5153.10	20.12	−3	0.0002

## Data Availability

Data available upon request from the corresponding author.

## Supplementary Materials

The following supporting information can be downloaded at: https://www.mdpi.com/article/10.3390/ani13223441/s1, Table S1: Days (*n* = 63) and hours (*n* = 287.8) of observation of bottlenose dolphins in CCSC–Aransas Pass, Texas, per month from June 2021 to September 2022; Table S2: Contingency table from the multiple logistic regression model of observed (top) and expected (middle) counts of each behavioral state across seasons (*n* = 4339 tracks) with chi-square value (bottom); Table S3: Contingency table from the multiple logistic regression R model of observed (top) and expected (middle) counts of each behavioral state across time of day (*n* = 4339 tracks) with chi-square value (bottom); Table S4: Contingency table from the multiple logistic regression model of observed (top) and expected (middle) counts of each behavioral state across vessel type (*n* = 4339 tracks) with chi-square value (bottom); Table S5: Contingency table from the multiple logistic regression model of observed (top) and expected (middle) counts of each behavioral state across vessel size (*n* = 4339 tracks) with chi-square value (bottom).

## References

[1] Culloch R.M., Anderwald P., Brandecker A., Haberlin D., McGovern B., Pinfield R., Visser F., Jessopp M., Cronin M. (2016). Effect of construction-related activities and vessel traffic on marine mammals. Mar. Ecol. Prog. Ser..

[2] Marley S.A., Kent C.P.S., Erbe C. (2017). Occupancy of bottlenose dolphins (*Tursiops aduncus*) in relation to vessel traffic, dredging, and environmental variables within a highly urbanized estuary. Hydrobiologia.

[3] Piwetz S. (2019). Common bottlenose dolphin (*Tursiops truncatus*) behavior in an active narrow seaport. PLoS ONE.

[4] Lusseau D. (2003). Effects of tour boats on the behavior of bottlenose dolphins: Using Markov chains to model anthropogenic impacts. Conserv. Biol..

[5] Schoeman R.P., Patterson-Abrolat C., Plön S. (2020). A global review of vessel collisions with marine animals. Front. Mar. Sci..

[6] Koroza A., Evans P.G. (2022). Bottlenose dolphin responses to boat traffic affected by boat characteristics and degree of compliance to code of conduct. Sustainability.

[7] Pirotta E., Laesser B.E., Hardaker A., Riddoch N., Marcoux M., Lusseau D. (2013). Dredging displaces bottlenose dolphins from an urbanized foraging patch. Mar. Pollut. Bull..

[8] Todd V.L.G., Todd I.B., Gardiner J.C., Morrin E.C.N., MacPherson N.A., DiMarzio N.A., Thomsen F. (2015). A review of impacts of marine dredging activities on marine mammals. ICES J. Mar. Sci..

[9] van Ginkel C., Becker D.M., Gowans S., Simard P. (2018). Whistling in a noisy ocean: Bottlenose dolphins adjust whistle frequencies in response to real-time ambient noise levels. Bioacoustics.

[10] Creel L. (2003). Ripple Effects: Population and Coastal Regions.

[11] Chilvers B.L., Lawler I.R., Macknight F., Marsh H., Noad M., Paterson R. (2005). Moreton Bay, Queensland, Australia: An example of the co-existence of significant marine mammal populations and large-scale coastal development. Biol. Conserv..

[12] Wells R.S., Scott M.D., Würsig B., Thewissen J.G.M., Kovacs K.M. (2018). Bottlenose dolphin, *Tursiops truncatus*, common bottlenose dolphin. Encyclopedia of Marine Mammals.

[13] Toth J.L., Hohn A.A., Able K.W., Gorgone A.M. (2011). Patterns of seasonal occurrence, distribution, and site fidelity of coastal bottlenose dolphins (*Tursiops truncatus*) in southern New Jersey, U.S.A. Mar. Mammal Sci..

[14] Lusseau D. (2004). The hidden cost of tourism: Detecting long-term effects of tourism using behavioral information. Ecol. Soc..

[15] Papale E., Azzolin M., Giacoma C. (2011). Vessel traffic affects bottlenose dolphin (*Tursiops truncatus*) behavior in waters surrounding Lampedusa Island, South Italy. J. Mar. Biol. Assoc. UK.

[16] Rivard A.E., Gelwick F.P., von Zharen W. (2016). Behavioral patterns of common bottlenose dolphins (*Tursiops truncatus*) within the Galveston-Port Bolivar ferry lane. Southeast. Nat..

[17] Arcangeli A., Crosti R. (2009). The short-term impact of dolphin-watching on the behavior of bottlenose dolphins (*Tursiops truncatus*) in western Australia. JMATE.

[18] Bejder L., Samuels A., Whitehead H., Gales N., Mann J., Connor R., Heithaus M., Watson-Capps J., Flaherty C., Krützen M. (2006). Decline in relative abundance of bottlenose dolphins exposed to long-term disturbance. Conserv. Biol..

[19] Mattson M.C., Thomas J.A., St. Aubin D. (2005). Effects of boat activity on the behavior of bottlenose dolphins (*Tursiops truncatus*) in waters surrounding Hilton Head Island, South Carolina. Aquat. Mamm..

[20] Vergara-Peña A. (2020). Effects of Marine Recreation on Bottlenose Dolphins in Cardigan Bay. Ph.D. Thesis.

[21] Würsig B., Lynn S.K., Jefferson T.A., Mullin K.E. (1998). Behavior of cetaceans in the northern Gulf of Mexico relative to survey ships and aircraft. Aquat. Mamm..

[22] Nowacek S.M., Wells R.S., Solow A.R. (2001). Short-term effects of boat traffic on bottlenose dolphins, *Tursiops truncatus*, in Sarasota Bay, Florida. Mar. Mammal Sci..

[23] Steckenreuter A., Harcourt R., Möller L. (2012). Are speed restriction zones an effective management tool for minimizing impacts of boats on dolphins in an Australian marine park?. Mar. Policy.

[24] Swim with and Approach Regulation for Hawaiian Spinner Dolphins under the Marine Mammal Protection Act, Volume 86 50 C.F.R. § Part 216. https://www.fisheries.noaa.gov/action/final-rule-prohibit-swimming-and-approaching-hawaiian-spinner-dolphins.

[25] PCCA Press Release (2023). Port of Corpus Christi Finishes Fiscal Year 2022 with Record Tonnage. https://portofcc.com/port-of-corpus-christi-finishes-fiscal-year-2022-with-record-tonnage/.

[26] PCCA Press Release (2020). Port of Corpus Christi Closes 2019 with Record Tonnage. https://portofcc.com/port-of-corpus-christi-closes-2019-with-record-tonnage/.

[27] U.S. Coast Guard (2019). Ports and Waterways Safety Assessment: Workshop Report Corpus Christi, Texas. https://www.navcen.uscg.gov/pdf/pawsa/WorkshopReports/Corpus_Christi_Sep_2019.pdf.

[28] Hrvacevic Z. (2022). Funding Secured for Phase 4 of the Corpus Christi Dredging Project. Dredging Today. https://www.dredgingtoday.com/2022/12/27/funding-secured-for-phase-4-of-the-corpus-christi-dredging-project/.

[29] Shane S.H. (1977). The Population Biology of the Atlantic Bottlenose Dolphin, *Tursiops truncatus*, in the Aransas Pass Area of Texas. Master’s Thesis.

[30] Shane S.H. (1980). Occurrence, movements, and distribution of bottlenose dolphin, *Tursiops truncatus*, in southern Texas. Fish. Bull..

[31] Leatherwood S., Reeves R.R. (1983). Abundance of bottlenose dolphins in Corpus Christi Bay and coastal Southern Texas. Contrib. Mar. Sci..

[32] NOAA National Ocean Service Tides and Currents [Data Set]. https://tidesandcurrents.noaa.gov/map/index.html.

[33] Harzen S.E. (2002). Use of an electronic theodolite in the study of movements of the bottlenose dolphin (*Tursiops truncatus*) in the Sado Estuary, Portugal. Aquat. Mamm..

[34] Azzellino A., Gaspari S., Airoldi S., Nani B. (2008). Habitat use and preferences of cetaceans along the continental slope and the adjacent pelagic waters in the western Ligurian Sea. Deep Sea Res. Part I Oceanogr. Res. Pap..

[35] Shane S.H., Leatherwood S., Reeves R.R. (1990). Behavior and ecology of the bottlenose dolphin at Sanibel Island, Florida. The Bottlenose Dolphin.

[36] Sagnol O., Reitsma F., Richter C., Field L.H. (2014). Correcting positional error in shore-based theodolite measurements of animals at sea. J. Mar. Biol..

[37] Henderson E.E., Würsig B. (2007). Behavior patterns of bottlenose dolphins in San Luis Pass, Texas. Gulf Mex. Sci..

[38] Torres L.G., Read A.J. (2009). Where to catch a fish? The influence of foraging tactics on the ecology of bottlenose dolphins (*Tursiops truncatus*) in Florida Bay, Florida. Mar. Mammal Sci..

[39] Shane S.H., Wells R.S., Würsig B. (1986). Ecology, behavior, and social organization of the bottlenose dolphin: A review. Mar. Mammal Sci..

[40] Baker I., O’Brien J., McHugh K., Berrow S. (2017). An ethogram for bottlenose dolphins (*Tursiops truncatus*) in the Shannon Estuary, Ireland. Aquat. Mamm..

[41] Upadhyay A. Formula to Find Bearing or Heading Angle between Two Points: Latitude Longitude. https://www.igismap.com/formula-to-find-bearing-or-heading-angle-between-two-points-latitude-longitude/.

[42] Marine Traffic Vessels database. AIS Marine Traffic: Global Ship Tracking Intelligence.

[43] Hua C., Choi Y., Shi Q. (2021). Companion to BER 643: Advanced Regression Methods.

[44] Félix F., Fernández J.E., Paladines A., Centeno R., Romero J., Burneo S.F. (2022). Habitat use of the common bottlenose dolphin (*Tursiops truncatus*) in the Gulf of Guayaquil, Ecuador: Management needs for a threatened population. Ocean Coast. Manag..

[45] McNulty K. (2021). Handbook of Regression Modeling in People Analytics.

[46] Shukla A. Multinomial Logistic Regression in R [Data Set]. https://rpubs.com/Anupam/588952.

[47] Pipis G. (2020). Contingency Tables in R. R-Bloggers. https://www.r-bloggers.com/2020/12/contingency-tables-in-r/.

[48] Wood S.N. (2001). Mgcv: GAMs and generalized ridge regression for R. R News.

[49] Wood S.N. (2006). Generalized Additive Models: An Introduction with R.

[50] Zuur A.F., Ieno E.N., Walker N.J., Saveliev A.A., Smith G.M. (2009). Mixed Effects Models and Extensions in Ecology with R.

[51] Jackson S. (2023). Machine Learning: Generalized Additive Models [Lecture Notes].

[52] Wedderburn R.W.M. (1974). Quasi-likelihood functions, generalized linear models, and Gauss-Newton method. Biometrika.

[53] Stokes G.M. (1977). Life history studies of Southern flounder (*Paralichthys lethostigma*) and Gulf flounder (*P. albigutta*) in the Aransas Bay area of Texas. TPWD.

[54] Bushon A. (2006). Recruitment, Spatial Distribution, and Fine-Scale Movement Patterns of Estuarine Dependent Species through Tidal Inlets in Texas. Ph.D. Thesis.

[55] Payne L.M. (2011). Evaluation of Large-Scale Movement Patterns of Spotted Seatrout (*Cynoscion nebulosus*) Using Acoustic Telemetry. Master’s Thesis.

[56] Rossbach K.A. (1999). Cooperative feeding among bottlenose dolphins (*Tursiops truncatus*) near Grand Bahamas Island, Bahamas. Aquat. Mamm..

[57] Nañez-James S.E., Stunz G.W., Holt S.A. (2009). Habitat use patterns of newly settled southern flounder (*Paralichthys lethostigma*) in Aransas-Copano Bay, Texas. Estuaries Coast.

[58] Mattos P.H., Rosa L.D., Fruet P.F. (2007). Activity budgets and distribution of bottlenose dolphins (*Tursiops truncatus*) in the Patos Lagoon estuary, southern Brazil. Lat. Am. J. Aquat. Mamm..

[59] Mate B.R., Rossbach K.A., Nieukirk S.L., Wells R.S., Irvine A.B., Scott M.D., Read A.J. (1995). Satellite-monitored movements and dive behavior of a bottlenose dolphin (*Tursiops truncatus*) in Tampa Bay, Florida. Mar. Mammal Sci..

[60] Bejder L., Samuels A., Whitehead H., Finn H., Allen S. (2009). Impact assessment research: Use and misuse of habituation, sensitization, and tolerance in describing wildlife responses to anthropogenic stimuli. Mar. Ecol. Prog. Ser..

[61] Higham J.E.S., Shelton E.J. (2011). Tourism and wildlife habituation: Reduced population fitness or cessation of impact?. Tour. Manag..

[62] Constantine R., Brunton D.H., Dennis T. (2004). Dolphin-watching tour boats change bottlenose dolphin (*Tursiops truncatus*) behavior. Biol. Conserv..

