# Supplementary material for: Vessels Disturb Bottlenose Dolphin Behavior and Movement in an Active Ship Channel
Source: Animals (Basel). 2023 Nov 8;13(22):3441. doi: 10.3390/ani13223441 (PMC10668690; doi:10.3390/ani13223441)
Supplement: Supplementary file 1 [file animals-13-03441-s001.zip › animals-2645287-supplementary.pdf]

Supplemental Materials

Table S1. Days (n = 63) and hours (n = 287.8) of observation of bottlenose dolphins in CCSC-Aransas Pass, Texas per month from June 2021 to September 2022.

Season	Month	Days/Month	Hours	Mean hours/day (± SD)
Summer	June	7	28.9	4.1 ± (1.4)
	July	10	47.2	4.7 ± (1.6)
	August	12	48.5	4.0 ± (1.5)
Fall	September	6	27.3	4.6 ± (0.9)
	October	3	14.2	4.7 ± (0.3)
	November	5	20.2	4.0 ± (0.9)
Winter	December	5	21.2	4.2 ± (0.9)
	January	2	9.1	4.6 ± (1.0)
	February	2	10.4	5.2 ± (0.7)
Spring	March	2	8.9	4.4 ± (1.0)
	April	2	11.5	5.7 ± (1.2)
	May	7	40.4	5.8 ± (0.4)

Table S2. Contingency table from the multiple logistic regression model of observed (top) and expected (middle) counts of each behavioral state across season (n = 4,339 tracks) with chi-square value (bottom). Star (*) indicates a chi-square value above model chi-square of 16.92.

Contingency Table		Season				
		Summer	Fall	Spring	Winter	Total
Behavior	Travel	564<br>485.19<br>12.80	302<br>260.52<br>6.61	174<br>247.27<br>21.71*	87<br>134.02<br>16.50	1127
	Forage	679<br>804.63<br>19.62*	424<br>432.04<br>0.15	420<br>410.07<br>0.24	346<br>222.26<br>68.88*	1869
	Mill	399<br>284.14<br>46.43*	104<br>152.57<br>15.46	111<br>144.81<br>7.89	46<br>78.49<br>13.45	660
	Social	226<br>294.041<br>15.74	173<br>157.8818<br>1.45	247<br>149.8539<br>62.98*	37<br>81.22332<br>24.08*	683
	Total	1868	1003	952	516	4339

Table S3. Contingency table from the multiple logistic regression R model of observed (top) and expected (middle) counts of each behavioral state across time of day (n = 4,339 tracks) with chi-square value (bottom). Star (*) indicates a chi-square value above model chi-square of 16.92.

Contingency Table		Time of Day				
		Mid-Day	Early Afternoon	Late Afternoon	Morning	Total
Behavior	Travel	426<br>420.00<br>0.09	492<br>392.72<br>25.10*	52<br>106.75<br>28.08*	157<br>207.53<br>12.30	1127
	Forage	672<br>696.51<br>0.86	530<br>651.29<br>22.59*	239<br>177.09<br>21.64*	428<br>344.27<br>20.36*	1869
	Mill	281<br>245.96<br>4.99	198<br>229.99<br>4.45	55<br>62.52<br>0.90	126<br>121.53<br>0.16	660
	Social	238<br>254.53<br>1.07	292<br>238.00<br>12.25	65<br>64.69532<br>0.001	88<br>125.77<br>11.34	683
	Total	1617	1512	411	799	4339

Table S4. Contingency table from the multiple logistic regression model of observed (top) and expected (middle) counts of each behavioral state across vessel type (n = 4,339 tracks) with chi-square value (bottom).

Contingency Table		Behavior				
		Travel	Forage	Mill	Social	Total
Vessel Type	None	295<br>229.35<br>18.79	363<br>380.35<br>0.79	98<br>134.31<br>9.82	127<br>138.99<br>1.035	883
	Coastal Cargo	33.00<br>27.01<br>1.33	29.00<br>44.80<br>5.57	30.00<br>15.82<br>12.71	12.00<br>16.37<br>1.17	104
	Ecotour	56.00<br>51.69<br>0.36	52.00<br>85.72<br>13.26	42.00<br>30.27<br>4.55	49.00<br>31.32<br>9.97	199
	Ferry	37<br>43.64<br>1.01	119<br>72.37<br>30.05	7<br>25.55<br>13.47	5<br>26.44<br>17.39	168
	Fishing	33<br>35.06<br>0.12	69<br>58.15<br>2.02	17<br>20.53<br>0.61	16<br>21.25<br>1.30	135
	Mixed	354<br>437.66<br>15.99	777<br>725.80<br>3.61	280<br>256.30<br>2.19	274<br>265.24<br>0.29	1685
	Offshore Commercial	19<br>14.54<br>1.36	24<br>24.12<br>0.001	8<br>8.52<br>0.032	5<br>8.81<br>1.65	56
	Personal	279	401	170	175	1025

		266.23<br>0.61	441.51<br>3.72	155.91<br>1.27	161.34<br>1.16	
	Enforcement	3<br>8.57<br>3.62	19<br>14.21<br>1.61	3<br>5.02<br>0.81	8<br>5.19<br>1.52	33
	Supply	9<br>7.79<br>0.19	7<br>12.92<br>2.71	4<br>4.56<br>0.07	10<br>4.72<br>5.89	30
	Tug	9<br>5.45<br>2.30	9<br>9.05<br>0.0002	1<br>3.19<br>1.51	2<br>3.31<br>0.52	21
	Total	1127	1869	660	683	4339

Table S5. Contingency table from the multiple logistic regression model of observed (top) and expected (middle) counts of each behavioral state across vessel size (n = 4,339 tracks) with chi-square value (bottom).

Contingency Table		Vessel Size						
		None	Extra Large	Large	Medium	Mixed	Small	Total
Behavior	Travel	295<br>229.3<br>18.8	27<br>22.3<br>1.0	19<br>18.4<br>0.0	62<br>64.2<br>0.1	358<br>423.1<br>10.0	366<br>369.6<br>0.0	1127
	Forage	363<br>380.3<br>0.8	34<br>37.0<br>0.3	39<br>30.6<br>2.3	107<br>106.4<br>0.0	785<br>701.7<br>9.9	541<br>612.9<br>8.4	1869
	Mill	98<br>134.3<br>9.8	17<br>13.1<br>1.2	5<br>10.8<br>3.1	45<br>37.6<br>1.5	240<br>247.8<br>0.2	255<br>216.5<br>6.9	660
	Social	127<br>139.0<br>1.0	8<br>13.5<br>2.3	8<br>11.2<br>0.9	33<br>38.9<br>0.9	246<br>256.4<br>0.4	261<br>224.0<br>6.1	683
	Total	883	86	71	247	1629	1423	4339
